# Estradiol mediates sex differences in aversion-resistant alcohol intake

**DOI:** 10.3389/fnins.2023.1282230

**Published:** 2023-11-02

**Authors:** Miranda E. Arnold, Ellie B. Decker Ramirez, Lauren A. Beugelsdyk, M. Vitória Siano Kuzolitz, Qiuyun Jiang, Jesse R. Schank

**Affiliations:** Department of Physiology and Pharmacology, College of Veterinary Medicine, University of Georgia, Athens, GA, United States

**Keywords:** aversion, alcohol, hormones, sex differences, ventral tegmental area

## Abstract

**Introduction:**

Alcohol consumption despite negative consequences is a core symptom of alcohol use disorder. This can be modeled in mice by pairing aversive stimuli with alcohol consumption, such as adding the bitter tastant quinine to the alcohol solution. If an animal continues to drink alcohol despite such negative stimuli, this is typically considered aversion-resistant, or inflexible, drinking behavior. Previous studies in our lab have found that females are more aversion-resistant than males in that they tolerate higher concentrations of quinine before they suppress their alcohol intake. Interestingly, we did not observe any differences in intake across the estrous cycle. In regards to neuronal activation patterns during quinine-alcohol intake, we have found that male mice show higher levels of activation in the ventromedial prefrontal cortex and posterior insular cortex, while females show higher levels of activation in the ventral tegmental area.

**Methods:**

In the experiments presented here, we conducted ovariectomies to further examine the role of circulating sex hormones in aversion-resistant alcohol intake and neuronal activation patterns. Furthermore, we used hormonal addback of estradiol or progesterone to determine which ovarian sex hormone mediates aversion-resistant consumption.

**Results:**

We found that ovariectomy reduced quinine-adulterated alcohol intake, demonstrating that circulating sex hormones play a role in this behavior. We also observed reduced neuronal activation in the VTA of ovariectomized mice compared to sham females, and that estradiol supplementation reversed the effect of ovariectomy on quinine-alcohol intake.

**Discussion:**

Taken together with our prior data, these findings suggest that circulating estradiol contributes to the expression of aversion-resistant alcohol intake and neuronal activity in the VTA. However, since this behavior is not affected by the estrous cycle, we believe this is due to a threshold level of this hormone, as opposed to fluctuations that occur across the estrous cycle.

## Introduction

According to data collected by the National Institute on Alcohol Abuse and Alcoholism (2023), 14.8 million people in the United States were diagnosed with alcohol use disorder (AUD) at that time, approximately two thirds of which were male (National Center for Drug Abuse Statistics, NIAAA). While this is roughly a 2:1 ratio of men to women with AUD, the gender gap has been steadily narrowing over the last few decades as a result of more women being diagnosed while men remain around the same prevalence (White, 2020). For example, in the 1980s, the ratio of men to women with AUD was 5:1 (Keyes et al., 2010). Multiple factors may underlie this narrowing gender gap including changes in social norms and sex differences in coping styles with regard to mental illness (Carvalho et al., 2019; McCaul et al., 2019). Women with AUD experience a higher incidence of complicating factors such as an increased rate of liver disease, increased risk of breast cancer, and more cardiovascular problems compared to men (Bradley et al., 1998; Freudenheim, 2020; Piano et al., 2020). Even though prevalence of AUD is increasing in women, and they suffer complications at higher rates, clinical and preclinical studies have historically used only male subjects. However, recent initiatives by the National Institutes of Health and other major research organizations are helping to reverse this trend. It is now widely acknowledged that it is essential to examine the role of sex when identifying contributing factors to the development of AUD, and much work needs to be done in this realm.

Compulsive alcohol intake is typified by the continuation of alcohol use despite negative consequences and is represented by several AUD criteria in the Diagnostic and Statistical Manual of Mental Disorders (DSM-5). Such consequences can include impaired relationships with friends and family, financial problems, poor work performance, legal trouble, exacerbation of existing physical/mental health conditions, and organ damage (Koob and Volkow, 2016; National Institute on Drug Abuse, 2023). Compulsive alcohol intake can be modeled in rodents by pairing aversive stimuli with alcohol consumption, such as delivering contingent footshock when alcohol is self-administered, or by adding the bitter tastant quinine to the alcohol solution (Seif et al., 2013; Randall et al., 2017; Fulenwider et al., 2019; Xie et al., 2019; Halladay et al., 2020; Sneddon et al., 2020; Katner et al., 2022; Arnold et al., 2023; Sneddon et al., 2023a). If the animal continues to consume the alcohol despite such negative consequences, this is considered aversion-resistant, or inflexible, alcohol consumption (Hopf and Lesscher, 2014).

Multiple research groups have begun to examine sex differences in aversion-resistant alcohol intake and aversion-associated neurocircuitry. For example, our lab has found that females continue to consume quinine-adulterated alcohol at concentrations of this tastant where males significantly reduce their consumption (Fulenwider et al., 2019; Arnold et al., 2023). This agrees with other studies that have observed increased aversion-resistant alcohol intake in females (Sneddon et al., 2020; Martins de Carvalho et al., 2023), but it should be noted that some studies have not detected a sex difference in this measure (Sneddon et al., 2019; Bauer et al., 2021; Katner et al., 2022). These disparate findings may be due to differences in drinking model and duration of alcohol access (see Arnold and Schank, 2023 for review).

In our initial studies on quinine-alcohol intake in females, we found that neither baseline nor quinine-adulterated alcohol intake was affected by estrous cycle (Fulenwider et al., 2019). Other groups have examined the role of ovariectomy on alcohol consumption and found that removal of ovaries reduces alcohol intake (Ford et al., 2002; Rajasingh et al., 2007; Satta et al., 2018). Additional experiments found that estradiol restoration in ovariectomized mice rescues alcohol intake to the levels of intact females. Similarly to Fulenwider et al., other groups have monitored the estrous cycle in intact females during alcohol consumption and did not observe a difference at any part of the cycle (Roberts et al., 1998; Melón et al., 2017; Satta et al., 2018; Fulenwider et al., 2019). Sneddon and colleagues examined the role of chromosome and gonad regulation on alcohol intake using the four core genotypes mouse model and found mice with ovaries (either XX/Sry- or XY/Sry-) exhibited increased alcohol drinking (Sneddon et al., 2022), and XY/Sry- (XY genotype with ovaries) promoted quinine alcohol intake (Sneddon et al., 2023b). Overall, it appears that ovarian sex hormones influence alcohol intake and quinine-alcohol consumption, but fluctuations of sex hormones during the estrous cycle does not seem to have an effect. Taken together, these findings suggest that there is a threshold level of sex hormones that influence alcohol intake, and that this not affected by the peaks and fluctuations of hormones during the estrous cycle.

Some groups have examined the neurocircuitry that contributes to aversion-resistant alcohol intake. For example, Seif and colleagues found that the nucleus accumbens core, medial prefrontal cortex, and insular cortex all contribute to quinine-alcohol intake (Seif et al., 2013), suggesting that these regions promote aversion-resistant consumption. However, Chen and colleagues observed an increase in Fos activation in the insular cortex, and increased suppression of intake during quinine-adulterated alcohol exposure, in males compared to females. This group also observed that degradation of perineuronal nets, which increases glutamatergic output from the insular cortex, reduces quinine-alcohol consumption (Chen and Lasek, 2019). In line with these findings, Martins de Carvalho et al. detected lower Fos activation in females compared to males in the anterior insula after quinine-adulterated alcohol drinking (Martins de Carvalho et al., 2023). Most of the findings above agree with the results of our recent experiments, in which we observed increased Fos activation in the posterior insular cortex of male mice, which more readily suppress their alcohol intake when adulterated with quinine (Arnold et al., 2023). Another cortical region where we observed increased neuronal activation in males following quinine-alcohol exposure is the ventromedial prefrontal cortex (vmPFC; Arnold et al., 2023). This fits well with the conception of the vmPFC as region that provides top down suppression of alcohol intake under conditions that are paired with punishment (Halladay et al., 2020).

As stated above, estradiol has been shown to increase alcohol consumption, but progesterone seems to have opposing effects on intake (Finn, 2020). For example, chronic alcohol, which typically leads to increased intake, causes decreased levels of progesterone in male and female mice (Rachdaoui and Sarkar, 2017). The VTA seems to be a specific region where estradiol influences dopaminergic function and alcohol responses. For example, DA neurons in the VTA show increased excitability during estrus (Shanley et al., 2023). In regards to ethanol responses, Vandegrift and colleagues have observed that higher estradiol states lead to more sensitive ethanol excitation in dopaminergic neurons of the VTA (Vandegrift et al., 2017). Furthermore, this group found that ERα specifically modulated VTA response to ethanol, and inhibitors of both ERα and ERβ reduced binge-like drinking in females (Vandegrift et al., 2020). These results are supported by findings from Calipari and colleagues, who observed enhanced dopamine neuronal activity in the VTA during estrus and found cocaine to have increased rewarding effects and ability to inhibit dopamine transporter function in this phase of the cycle (Calipari et al., 2017). In line with these results, we have found that neuronal activity is increased in the VTA of females (which are more aversion-resistant) during the consumption of quinine-alcohol (Arnold et al., 2023).

To identify if ovarian sex hormones influence quinine-adulterated alcohol intake, we performed ovariectomies to remove circulating sex hormones. We aimed to identify if aversion-resistant alcohol consumption as well as the neuronal activity in the vmPFC, PIC, and VTA is influenced by this intervention. Following this, we used hormonal addback to dissect the effects of estradiol and progesterone on quinine-adulterated alcohol intake.

## Materials and methods

### Animals

Male and female C57BL/6 J mice (Jackson Labs) aged 8 to 10 weeks at the start of the study were used for these experiments. All mice were singly housed on a reverse 12:12 light/dark cycle. All experiments were in agreement with NIH guidelines and approved by University of Georgia Institutional Animal Care and Use Committee.

### Ovariectomy and hormone supplementation

Female mice were anesthetized by isoflurane (3.5%–4% induction and maintained at 2%). The lumbar dorsal area was shaved bilaterally, and mice were placed on a heating pad dorsal side up. A small incision was made to the skin approximately 1 centimeter from the midline of the back. Another small incision at the abdominal wall was made to access the para-ovarian fatty tissue. The para-ovarian fatty tissue was lifted and cut between the oviduct and ovary to remove the ovary. The fatty tissue and oviduct were placed back into the abdominal cavity. The abdominal wall was sutured with 4–0 Monocryl, absorbable sutures (Ethicon, LLC), and incision to the skin was closed with 4–0 prolene, non-absorbable suture (Ethicon, LLC). This was repeated for the other ovary. Metronidazole paste was applied to the incision sites to discourage licking. Sham surgeries were performed using the same procedure but without the removal of ovaries in separate groups of females and males. All mice were given subcutaneous carprofen (5 mg/kg) at the start of the surgery and 2 days post-operatively. Animals were able to recover for 10 to 14 days before two bottle choice procedure.

A separate cohort of females also received ovariectomies or sham surgeries, with the additional implantation of subcutaneous 90-day, slow releasing hormone pellets (Innovative Research of America). Pellets were implanted on the lateral side of the neck by making a small incision between the shoulder and ear and sutured with 4–0 prolene, non-absorbable sutures. Pellets contained 17β-estradiol 0.32 mg/pellet, progesterone 5 mg/pellet, or placebo (with no additional hormone composition). 17β-estradiol 0.32 mg/pellet releases about 32 pg./mL per day similar to average levels observed in C57BL/6 J mice (Ström et al., 2012). Progesterone 5 mg/pellet releases 5 mg of progesterone per day and was determined based on previous studies (Nelson et al., 1981; Sahores et al., 2013). All pellets are made up of a matrix of cholesterol, lactose, celluloses, phosphates, and stearates. Animals were able to recover 10 to 14 days before two bottle choice.

### Two bottle choice

Mice were singly housed and given 24-h access to two bottles: one with tap water and the other with 20% alcohol (v/v). Alcohol was diluted from 95% EtOH (United States Pharmacopeia, Decon Labs) in tap water. Bottles were weighed every 24 h at the onset of the dark cycle and converted into g/kg to measure consumption. Positions of bottles were switched daily. Mice were given 10 to 14 days of two bottle choice under these conditions to allow consumption rates to stabilize (less than 20% variability from the mean for 3 consecutive days). One sham surgery female was removed from the study due to low levels of drinking that were detected as an outlier using Grubbs test.

### Quinine-adulterated alcohol intake

After 10–14 days of alcohol consumption, 20% alcohol solution was adulterated with 0.1 mM quinine hydrochloride, based on our prior work (Fulenwider et al., 2019; Arnold et al., 2023). Mice were presented with this concentration 3 times with 2 days of quinine-free alcohol in between. Intake of the last 2 days of quinine-adulterated alcohol were averaged and converted to percent change from the individual animal’s baseline intake. Baseline consumption rate was calculated from the last 3 days of 20% alcohol access prior to the start of any quinine adulteration. Multiple quinine-alcohol exposure days helps to reduce the effect of neophobic taste response, and provides a stable, accurate assessment of aversion-related alcohol intake.

### Fos immunohistochemistry

To assess neuronal activation, an extra session of quinine-adulterated alcohol intake was conducted to examine Fos expression. These animals were provided with a water bottle and 0.1 mM quinine-alcohol bottle and allowed to drink for 2.5 h (1 h for alcohol intake and 1.5 h for Fos protein expression). Mice were then deeply anesthetized using ketamine (100 mg/kg) and xylazine (20 mg/kg) solution. Animals were intracardially perfused with 4% paraformaldehyde (PFA), brains were removed, transferred to 30% sucrose, and frozen on dry ice. Each brain was sectioned on a Leica cryostat at 30-micron thickness. Sections for the vmPFC (+1.54 mm bregma), PIC (−0.2 mm bregma), and VTA (−3.08 mm bregma) were stained for Fos protein, an immediate early gene, to identify neuronal activation. These regions were selected based on prior work from our lab showing sex differences in Fos signal (Arnold et al., 2023). Briefly, sections were blocked with Normal Donkey Serum (Invitrogen), and then incubated using 1:1,000 rabbit anti-cFos (Cell Signaling Technology #2250) for 72 h at 4°C. Following primary antibody, the tissue was then incubated using undiluted ImmPress Horse Anti-Rabbit IgG (Vector Laboratories 30,026) at room temperature for 2 h and washed twice in PBS-Triton X-100 (0.3%) and once in 1× PBS for 10 min. Sections were then incubated in diaminobenzidine (DAB) substrate (Vector Laboratories SK-4100) for 3.5 min for vmPFC, and 10 min for PIC and VTA. Tissue was mounted on gelatin-coated microscope slides and dried overnight. Microscope slides then went through a dehydration series, xylene clearing, and were cover slipped. One brain section per subject (*n* = 9–12/group) was imaged on a Zeiss Axioscope A1 at 40x. Images were scored using ImageJ by a experimenter blind to treatment group.

### Statistics

Analysis was performed on GraphPad Prism (Version 9.5.1). To analyze baseline 20% alcohol intake, aversion resistant alcohol intake, and Fos neuronal activation, one-way ANOVAs were utilized to compare treatment groups, followed by *Bonferroni post hoc* analysis. Two-way ANOVA was used to compare treatment groups across days. *p*-values < 0.05 were considered significant.

## Results

### Ovariectomy affects baseline and aversion-resistant alcohol intake

**Figure 1:**
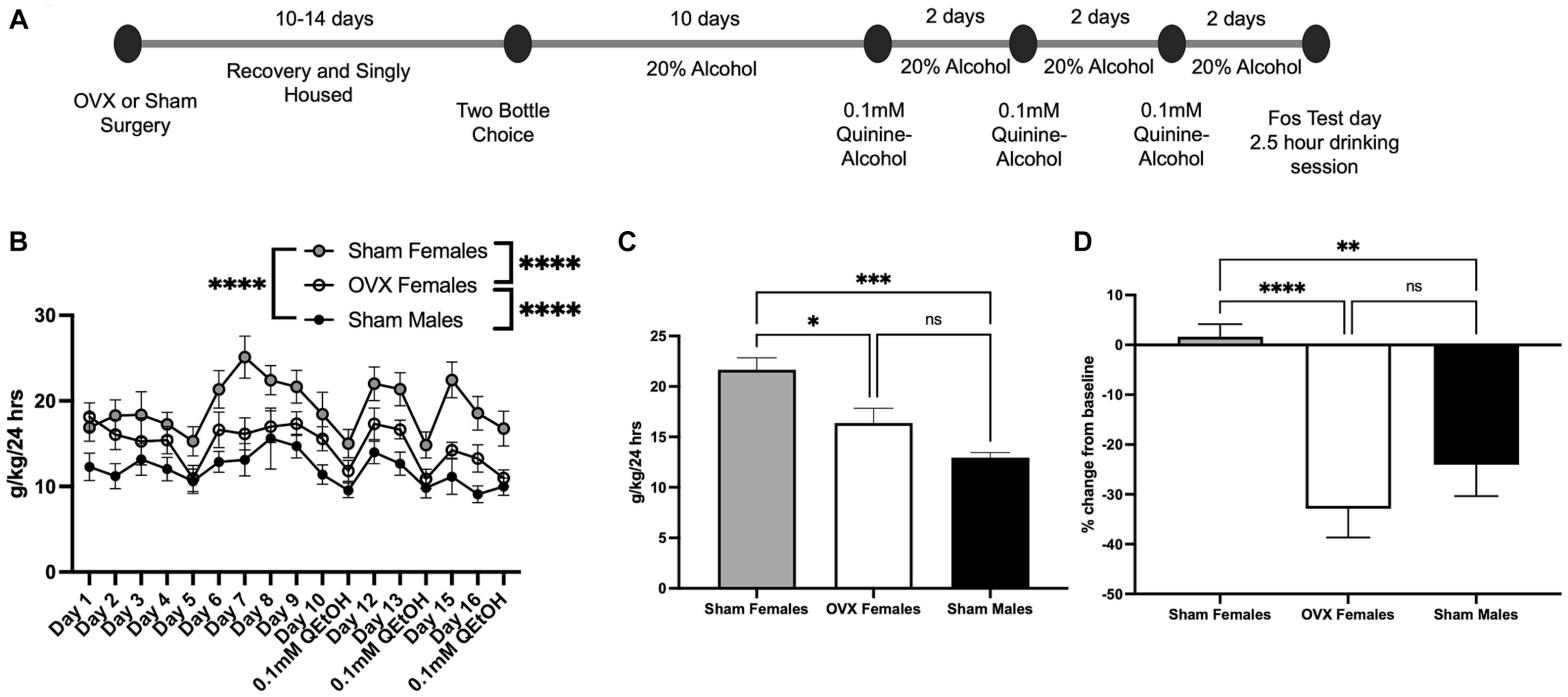
Ovariectomy impacts baseline and aversion resistant alcohol intake. **(A)** Timeline for drinking and quinine adulteration. **(B)** Consumption rate over all days of intake including quinine-adulterated alcohol days. **(C)** Average of last 3 days of baseline 20% alcohol in sham females, OVX females, and sham males. **(D)** Aversion resistant alcohol intake measured as percent change from the animals’ individual baseline for sham females, OVX females, and sham males. *p < 0.05, **p < 0.01, ***p < 0.001, ****p < 0.0001.

Ovariectomized female (OVX; *n* = 12), sham surgery female (*n* = 11), and sham surgery male mice (*n* = 9) were allowed to consume 20% alcohol for 10 days, with the last 3 days averaged to calculate baseline alcohol intake (Figure 1A). Across all days of drinking, two-way ANOVA analysis detected a main effect of day [*F*(16,494) = 4.56, *p* = 0.004; Figure 1B] and treatment group [*F*(2,494) = 67.4, *p* < 0.0001]. The day × treatment interaction effect did not reach significance [*F*(32,494) = 0.67. *p* = 0.91]. *Bonferroni post hoc* analysis across the factor of treatment group indicated that sham females showed the highest level of alcohol intake (sham females vs. OVX females: *p* < 0.0001, sham females vs. sham males: *p* < 0.0001). OVX females exhibited an intermediate consumption rate, differing from both sham females (*p* < 0.0001, see above) and sham males (*p* < 0.0001). The average of the last 3 days of alcohol consumption was analyzed using one-way ANOVA, which identified a main effect of treatment group [Figure 1C, *F*(2,29) = 12.4, *p* = 0.0001]. *Bonferroni post hoc* analysis indicated a significant difference between sham male and sham female alcohol consumption (*p* = 0.0001) and between OVX females and sham females (*p* = 0.01), with sham females consuming more alcohol than the other two groups. OVX females and sham males did not differ (*p* = 0.18).

To assess aversion-resistant alcohol intake, 20% alcohol was adulterated with 0.1 mM quinine hydrochloride. Previously, our group observed a sex difference at this concentration of quinine-alcohol (Arnold et al., 2023). Similar to our previous studies, sham females exhibited aversion-resistance when presented with 0.1 mM quinine-alcohol in that they did not reduce their intake relative to baseline (Figure 1D). One-way ANOVA revealed a significant effect of treatment group [*F*(2,29) = 13.1, *p* < 0.0001]. *Bonferroni post hoc* test revealed that both males (*p* = 0.005) and OVX females (*p* < 0.0001) showed significantly lower quinine-alcohol intake compared to sham females. OVX females and sham males did not differ (*p =* 0.71). These results suggest that circulating ovarian hormones have a role in regulating quinine-adulterated alcohol intake.

### Ovariectomy impacts neuronal activation during quinine alcohol consumption

Our prior work identified 3 brain regions that exhibit a sex difference during aversion resistant alcohol consumption (Arnold et al., 2023). Specifically, we have found that males show higher neuronal activation in the vmPFC and PIC when consuming quinine adulterated alcohol. In contrast, females show higher neuronal activation in the VTA. To explore the role of circulating ovarian hormones on neuronal activation in these 3 brain regions, brains were obtained for Fos staining after quinine-adulterated alcohol intake.

**Figure 2:**
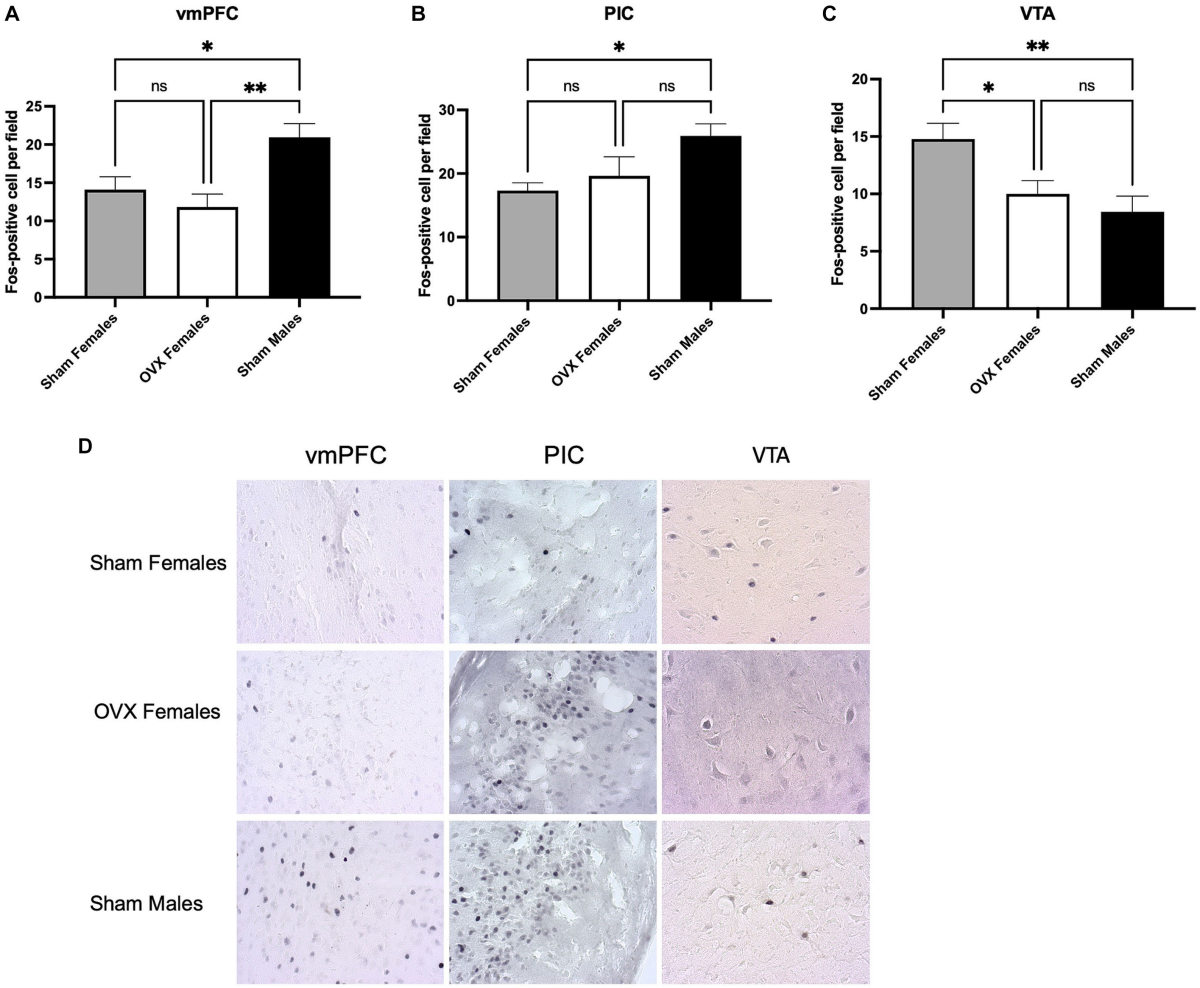
Impact of circulating ovarian hormones on neuronal activation. **(A)** Fos counts in the vmPFC shows higher neuronal activation in sham males compared to OVX and sham females. **(B)** Neuronal activation was higher in the PIC in sham males compared to sham females. **(C)** OVX females and sham males have lower neuronal activity in the VTA compared to sham females. **(D)** Representative images of Fos DAB immunohistochemistry. ^*^p < 0.05, ^**^p < 0.01, ^***^p < 0.001.

Similar to our previous study, sham males exhibited higher neuronal activation in the vmPFC [Figure 2A; one-way ANOVA: *F*(2,29) = 6.4, *p* = 0.005]. *Bonferroni post hoc* analysis revealed a difference in Fos positive neurons between sham males and both sham females (*p* = 0.04) and OVX females (*p* = 0.004). No differences between sham females and OVX females were detected (*p* > 0.99). The PIC also exhibited increased neuronal activation in sham males during quinine-adulterated alcohol consumption [Figure 2B; one-way ANOVA: *F*(2,30) = 3.6, *p* = 0.04]. *Bonferroni post hoc* analysis identified a significant difference in Fos positive neurons between sham males and sham females (*p* = 0.04), but the difference between sham males and OVX females did not reach significance (*p* = 0.19). There was also no significant difference between OVX females and sham females (*p* > 0.99). Together, these findings suggest that circulating ovarian hormones do not have a significant impact on neuronal activity in the vmPFC and the PIC during quinine-alcohol consumption.

In agreement with our previous findings, we observed increased neuronal activation in the VTA of sham female mice during quinine adulterated alcohol consumption [Figure 2C; one-way ANOVA: *F*(2,30) = 6.5, *p* = 0.005]. Specifically, sham females showed a significant increase in Fos positive neurons compared to sham males (*Bonferroni post hoc* analysis: *p* = 0.007). Interestingly, OVX females displayed lower neuronal activity compared to sham females (*p* = 0.03), and no differences were observed between sham males and OVX females (*p* > 0.999; Representative images of Fos immunostaining are shown in Figures 2D). These findings suggest that the sex differences in VTA activation are prevented by ovariectomy.

### Effects of hormonal supplementation on aversion-resistant intake in ovariectomized mice

**Figure 3:**
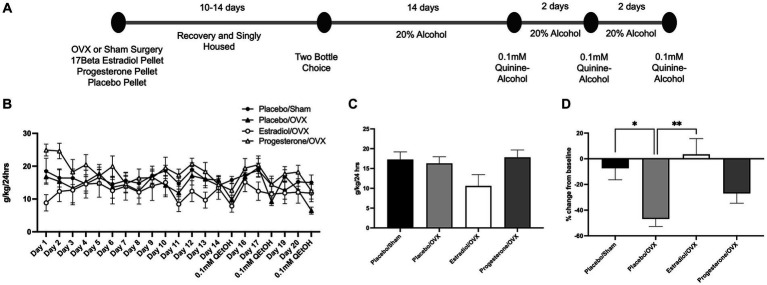
Estradiol rescues aversion resistant alcohol intake. **(A)** Timeline of alcohol consumption. **(B)** 20% alcohol consumption over 14 days was measured in four different treatment groups. **(C)** No difference was observed between groups during baseline alcohol intake. **(D)** Placebo/Sham females and OVX females that received estradiol treatment exhibited aversion resistant alcohol intake. ^*^p < 0.05, ^**^p < 0.01, ^***^p < 0.001.

Given that we observed a prevention of aversion-resistant alcohol intake following ovariectomy in female mice, we next aimed to assess if estrogen or progesterone restoration was able to rescue this behavior (Figure 3A). For this procedure, 4 groups of females were assessed for baseline and quinine-adulterated alcohol intake: OVX with a 17β estradiol pellet (Estradiol/OVX; *n* = 8), OVX with a progesterone pellet (Progesterone/OVX; *n* = 7), OVX with a placebo pellet (Placebo/OVX; *n* = 7), and sham surgery female with a placebo pellet (Placebo/Sham; *n* = 7). For alcohol consumption throughout the procedure, two-way ANOVA revealed main effect of day [Figure 3B; *F*(20,525) = 1.84, *p* = 0.015], and a main effect of treatment group [*F*(3,525) = 16.3, *p* < 0.0001], but did not identify an interaction between these factors [*F*(60,525) = 0.60, *p* = 0.99]. The average of the last 3 days of consumption prior to quinine-alcohol sessions was not significantly different between groups [Figure 3C, one-way ANOVA: *F*(3,25) = 2.4, *p* = 0.09].

During quinine-adulterated alcohol intake, one-way ANOVA revealed an effect of treatment group on percent change from baseline consumption [*F*(3,25) = 5.7, *p* = 0.004; Figure 3D]. In line with the results shown in Figure 1, Placebo/OVX females significantly reduced quinine-alcohol consumption while Placebo/sham females remained near their baseline alcohol intake (*Bonferroni post hoc* comparison between these groups: *p* = 0.04). The Progesterone/OVX group did not exhibit any differences between either the Placebo/Sham (*p* = 0.94) or the Placebo/OVX group (*p* = 0.90). Interestingly, the Estradiol/OVX group exhibited an aversion-resistant phenotype and showed less suppression of intake than the Placebo/OVX group (*p* = 0.004), and no difference from the Placebo/Sham (*p* > 0.99) group. This suggests that estrogen addback is able to rescue the aversion-resistant phenotype in ovariectomized female mice.

## Discussion

The primary findings reported here are that ovariectomy prevents the expression of aversion-resistant alcohol intake in female mice, and that this effect is rescued by exogenous addback of estradiol. Whether ovariectomy reduces alcohol intake at baseline is not entirely clear, as one experiment showed this effect and the other did not. However, a reduction in alcohol intake following ovariectomy would be consistent with prior work from other groups (Becker et al., 1985; Hilakivi-Clarke, 1996; Torres et al., 2014; Satta et al., 2018; Vandegrift et al., 2020). Importantly, we observed an impact of ovariectomy on quinine-alcohol intake in both experiments, and we used percent change from baseline to calculate aversion resistant alcohol consumption, correcting for any potential differences in baseline intake. This effect of ovariectomy was reversed by exogenous addback of estradiol but not progesterone. We also found that neuronal activation patterns during quinine-alcohol intake were altered by ovariectomy in the VTA, but not the vmPFC or PIC, 3 regions where we have previously observed sex differences in activation by quinine-alcohol (Arnold et al., 2023). We believe this is specific to aversion-related alcohol intake, and not general alcohol consumption, as our prior study did not observe any sex difference in neuronal activation in the VTA during quinine-free alcohol consumption. Taken together, these results suggest that sex differences in aversion-resistant alcohol intake and the associated activation of the VTA are mediated by circulating estradiol.

Our findings of sex differences in quinine-alcohol intake, with females being less sensitive to this aversive consequence during alcohol consumption, replicates prior studies from our group (Fulenwider et al., 2019; Arnold et al., 2023). Importantly, our published work has shown no sex difference in sensitivity to quinine-adulterated water, suggesting that this difference is not due to taste sensitivity. In Fulenwider et al., we also found that aversion-resistant alcohol intake in females was not affected by estrous cycle (Fulenwider et al., 2019). Thus, in the experiments presented here, our primary objective was to determine if this behavior in females is driven by organizational/developmental factors, or if threshold levels of circulating sex hormones are sufficient to establish these sex differences. Our ovariectomy experiments suggest that circulating ovarian hormones are critical, as this intervention prevented the expression of aversion-resistant intake. These findings contrast with recently published data from Sneddon and colleagues, who report that OVX females are more aversion resistant compared to intact females (Sneddon et al., 2023c). These discrepancies may be due to differences in drinking model (24-h access vs. drinking in the dark), the time in between quinine adulterated alcohol drinking sessions, or the amount of time the animals were able to recover from the ovariectomy procedure. Further analysis of these discrepancies should directly assess if different drinking models result in altered sensitivity to detect sex differences in aversion resistance.

Hormone supplementation allowed us to examine whether progesterone or estradiol more strongly mediates these effects, and we found that estradiol was the key player in establishing sex differences in aversion-resistant alcohol consumption. This role of estradiol in increased alcohol consumption generally agrees with prior work. For example, the addition of exogenous 17β-estradiol to ovariectomized females has been shown to increase alcohol consumption compared to OVX/placebo groups (Ford et al., 2002; Rajasingh et al., 2007; Torres et al., 2014; Satta et al., 2018). However, we did not observe an increase in alcohol consumption in our OVX/estradiol group compared to our OVX/placebo group. In fact, our estradiol treated mice appeared to drink less at baseline, although analysis of this measure did not reach statistical significance. The cause for this slight reduction is unknown, but it is important to highlight that we calculate aversion resistance as a relative change from baseline intake, which should account for any group variation in baseline consumption. Importantly, estradiol treated mice did not reduce their rates from baseline during quinine adulterated alcohol intake, suggesting that estradiol addback rescued aversion resistance. In contrast to the effects of estradiol, it has been shown that alcohol intake reduces progesterone levels (Sarkola et al., 1999; Gill, 2000; Finn, 2020), and progesterone levels negatively correlate with alcohol drinking (Weinland et al., 2021). In our experiments, progesterone addback induced intermediate levels of quinine-induced suppression of alcohol intake, and the difference between this group and sham/placebo group did not reach statistical significance. However, these mice did suppress their intake, and appeared similar to OVX/placebo mice. Taken together, these findings suggest a key role of estradiol in regulating aversion-resistant alcohol intake.

Prior research from our group has also identified a sex difference in neuronal activation patterns during quinine-adulterated alcohol consumption (Arnold et al., 2023). Specifically, males showed higher Fos positive cell counts in the vmPFC and PIC when exposed to quinine-adulterated alcohol, and females had higher Fos counts in the VTA under these conditions. Thus, it seems that these cortical areas provide top-down inhibition of alcohol seeking that is associated with negative stimuli, and the VTA drives alcohol intake that is aversion-resistant. In this study, we examined if removal of circulating sex hormones by ovariectomy would change neuronal activity in these areas. We found that ovariectomy did not affect neuronal activity in the vmPFC, with males showing higher levels of neuronal activation, and OVX females showing similar levels to sham females. In the PIC, males also exhibited increased neuronal activation compared to females; however, OVX females showed an intermediate level of activation between males and sham females, suggesting the possibility of circulating sex hormones partially contributing to quinine-alcohol induced neuronal activity in the PIC, but this effect appears to be weak at best.

In contrast to the effect of ovariectomy on cortical activation, we found a strong effect of OVX on Fos activation in the VTA, where OVX females had Fos positive cell counts similar to sham males, and much lower than sham females. This suggests that ovarian hormones impact VTA neuronal activity. While we did not directly assess the effect of hormonal addback on Fos activation, we would predict that estradiol restoration, which rescues the aversion-resistance phenotype, would also rescue neuronal activation patterns in the VTA. Other groups have examined estrogen regulation of dopamine signaling in the VTA (Vandegrift et al., 2017) and have found that during diestrus, VTA dopamine neurons were more sensitive to excitation by alcohol. This group also examined ERα and ERβ activity during the alcohol exposure, and found that ERα promoted the enhanced alcohol response (Vandegrift et al., 2020). Knockdown manipulations of both receptors resulted in reduced alcohol consumption in female but not male mice. These findings also agree with work showing increased excitability of DA neurons in the VTA and increased sensitivity to cocaine administration during the estrous phase in mice (Calipari et al., 2017; Shanley et al., 2023). Taken together with our data, these findings suggest that circulating estradiol contributes to alcohol seeking behavior and the associated activation of the VTA.

Overall, our results demonstrate increased aversion-resistant alcohol intake in female mice relative to males, and that this is likely mediated by circulating estradiol and its effect on VTA output. One outstanding question that will be addressed in current studies is what type of cells in the VTA are activated by alcohol and contribute to quinine-alcohol intake. This region consists primarily of dopaminergic and GABAergic neurons, and we will examine the specific activation patterns in these cell types during quinine-alcohol exposure. Additionally, it is unclear what drives sex differences in cortical activity, as ovariectomy had no impact on these measures. It is possible that male sex hormones promote the activity of these regions, and future experiments will examine this possibility. In conclusion, our study shows major sex differences in aversion-resistant behavior that is influenced by estradiol, and suggests that future work should further examine these mechanisms to improve the development of treatment strategies for women with AUD.

## Data availability statement

The raw data supporting the conclusions of this article will be made available by the authors, without undue reservation.

## Ethics statement

The animal study was approved by University of Georgia Institutional Animal Care and Use Committee. The study was conducted in accordance with the local legislation and institutional requirements.

## Author contributions

MA: Conceptualization, Data curation, Formal analysis, Investigation, Methodology, Writing – original draft, Writing – review & editing, Visualization. ED: Investigation, Writing – original draft. LB: Investigation, Writing – original draft. MS: Investigation, Writing – original draft. QJ: Investigation, Visualization, Writing – original draft. JS: Conceptualization, Data curation, Formal analysis, Funding acquisition, Methodology, Project administration, Resources, Supervision, Writing – original draft, Writing – review & editing.

## References

[ref1] Arnold M. E. Butts A. N. Erlenbach T. R. Amico K. N. Schank J. R. (2023). Sex differences in neuronal activation during aversion-resistant alcohol consumption. Alcohol. Clin. Exp. Res. 47, 240–250. doi: 10.1111/acer.15006, PMID: 36575056 PMC9992309

[ref2] Arnold M. E. Schank J. R. (2023). Aversion-associated drug and alcohol seeking in females. Front. Neuroendocrinol. 71:101095. doi: 10.1016/j.yfrne.2023.101095 37558185

[ref3] Bauer M. R. McVey M. M. Boehm S. L. (2021). Three weeks of binge alcohol drinking generates increased alcohol front-loading and robust compulsive-like alcohol drinking in male and female C57BL/6J mice. Alcohol. Clin. Exp. Res. 45, 650–660. doi: 10.1111/acer.14563, PMID: 33496972 PMC8443007

[ref4] Becker H. C. Anton R. F. De Trana C. Randall C. L. (1985). Sensitivity to ethanol in female mice: effects of ovariectomy and strain. Life Sci. 37, 1293–1300. doi: 10.1016/0024-3205(85)90244-9, PMID: 4046734

[ref5] Bradley K. A. Badrinath S. Bush K. Boyd-Wickizer J. Anawalt B. (1998). Medical risks for women who drink alcohol. J. Gen. Intern. Med. 13, 627–639. doi: 10.1046/j.1525-1497.1998.cr187.x, PMID: 9754520 PMC1497016

[ref6] Calipari E. S. Juarez B. Morel C. Walker D. M. Cahill M. E. Ribeiro E.. (2017). Dopaminergic dynamics underlying sex-specific cocaine reward. Nat. Commun. 8:13877. doi: 10.1038/ncomms13877, PMID: 28072417 PMC5234081

[ref7] Carvalho A. F. Heilig M. Perez A. Probst C. Rehm J. (2019). Alcohol use disorders. Lancet 394, 781–792. doi: 10.1016/S0140-6736(19)31775-1 31478502

[ref8] Chen H. Lasek A. W. (2019). Perineuronal nets in the insula regulate aversion-resistant alcohol drinking. Addict. Biol. 25:e12821. doi: 10.1111/adb.12821 31433552 PMC7032993

[ref9] Finn D. A. (2020). The endocrine system and alcohol drinking in females. Alcohol Res. 40:02. doi: 10.35946/arcr.v40.2.02, PMID: 32714716 PMC7374925

[ref10] Ford M. M. Eldridge J. C. Samson H. H. (2002). Ethanol consumption in the female long–Evans rat: a modulatory role of estradiol. Alcohol 26, 103–113. doi: 10.1016/S0741-8329(01)00203-8 12007585

[ref11] Freudenheim J. L. (2020). Alcohol’s effects on breast Cancer in women. Alcohol Res. 40, 1–12. doi: 10.35946/arcr.v40.2.11 PMC7295577 32582503

[ref12] Fulenwider H. D. Nennig S. E. Price M. E. Hafeez H. Schank J. R. (2019). Sex differences in aversion-resistant ethanol intake in mice. Alcohol Alcohol. 54, 345–352. doi: 10.1093/alcalc/agz022, PMID: 30888414

[ref13] Gill J. (2000). The effects of moderate alcohol consumption on female hormone levels and reproductive function. Alcohol Alcohol. 35, 417–423. doi: 10.1093/alcalc/35.5.417 11022013

[ref14] Halladay L. R. Kocharian A. Piantadosi P. T. Authement M. E. Lieberman A. G. Spitz N. A.. (2020). Prefrontal regulation of punished ethanol self-administration. Biol. Psychiatry 87, 967–978. doi: 10.1016/j.biopsych.2019.10.030, PMID: 31937415 PMC7217757

[ref15] Hilakivi-Clarke L. (1996). Role of estradiol in alcohol intake and alcohol-related behaviors. J. Stud. Alcohol 57, 162–170. doi: 10.15288/jsa.1996.57.162, PMID: 8683965

[ref16] Hopf F. W. Lesscher H. M. B. (2014). Rodent models for compulsive alcohol intake. Alcohol 48, 253–264. doi: 10.1016/j.alcohol.2014.03.001, PMID: 24731992 PMC4993047

[ref17] Katner S. N. Sentir A. M. Steagall K. B. Ding Z. M. Wetherill L. Hopf F. W.. (2022). Modeling aversion resistant alcohol intake in Indiana alcohol-preferring (P) rats. Brain Sci. 12:1042. doi: 10.3390/brainsci12081042, PMID: 36009105 PMC9406111

[ref18] Keyes K. M. Martins S. S. Blanco C. Hasin D. S. (2010). Telescoping and gender differences in alcohol dependence: new evidence from two national surveys. Am. J. Psychiatry 167, 969–976. doi: 10.1176/appi.ajp.2009.09081161, PMID: 20439391 PMC3767417

[ref19] Koob G. F. Volkow N. D. (2016). Neurobiology of addiction: a neurocircuitry analysis. Lancet Psychiatry 3, 760–773. doi: 10.1016/S2215-0366(16)00104-8, PMID: 27475769 PMC6135092

[ref20] Martins de Carvalho L. Chen H. Sutter M. Lasek A. W. (2023). Sexually dimorphic role for insular perineuronal nets in aversion-resistant alcohol consumption. Front. Psych. 14:1122423. doi: 10.3389/fpsyt.2023.1122423 PMC10011443 36926460

[ref21] McCaul M. E. Roach D. Hasin D. S. Weisner C. Chang G. Sinha R. (2019). Alcohol and women: a brief overview. Alcohol. Clin. Exp. Res. 43, 774–779. doi: 10.1111/acer.13985, PMID: 30779446 PMC6502688

[ref22] Melón L. C. Nolan Z. T. Colar D. Moore E. M. Boehm S. L. (2017). Activation of extrasynaptic δ-GABAA receptors globally or within the posterior-VTA has estrous-dependent effects on consumption of alcohol and estrous-independent effects on locomotion. Horm. Behav. 95, 65–75. doi: 10.1016/j.yhbeh.2017.07.015, PMID: 28765080 PMC5623082

[ref23] National Institute on Alcohol Abuse and Alcoholism (2023). Alcohol use in the United States: age groups and demographic characteristics National Institute on Alcohol Abuse and Alcoholism. Available at: https://www.niaaa.nih.gov/.

[ref24] National Institute on Drug Abuse (2023), NIDA. Available at: https://drugabusestatistics.org/.

[ref25] Nelson J. F. Felicio L. S. Osterburg H. H. Finch C. E. (1981). Altered profiles of estradiol and progesterone associated with prolonged estrous cycles and persistent vaginal cornification in aging C57BL/6J mice. Biol. Reprod. 24, 784–794. doi: 10.1095/biolreprod24.4.784, PMID: 7195743

[ref26] Piano M. R. Thur L. A. Hwang C. L. Phillips S. A. (2020). Effects of alcohol on the cardiovascular system in women. Alcohol Res. 40, 1–9. doi: 10.35946/arcr.v40.2.12 PMC7398617 32766021

[ref27] Rachdaoui N. Sarkar D. K. (2017). Pathophysiology of the effects of alcohol abuse on the endocrine system. Alcohol Res. 38, 255–276. PMID: 28988577 10.35946/arcr.v38.2.08 PMC5513689

[ref28] Rajasingh J. Bord E. Qin G. Ii M. Silver M. Hamada H.. (2007). Enhanced voluntary alcohol consumption after estrogen supplementation negates estrogen-mediated vascular repair in Ovariectomized mice. Endocrinology 148:3618. doi: 10.1210/en.2006-1357 17478555

[ref29] Randall P. A. Stewart R. T. Besheer J. (2017). Sex differences in alcohol self-administration and relapse-like behavior in long-Evans rats. Pharmacol. Biochem. Behav. 156, 1–9. doi: 10.1016/j.pbb.2017.03.005, PMID: 28347737 PMC5494833

[ref30] Roberts A. J. Smith A. D. Weiss F. Rivier C. Koob G. F. (1998). Estrous cycle effects on operant responding for ethanol in female rats. Alcohol. Clin. Exp. Res. 22, 1564–1569. doi: 10.1111/j.1530-0277.1998.tb03950.x, PMID: 9802543

[ref31] Sahores A. Luque G. M. Wargon V. May M. Molinolo A. Becu-Villalobos D.. (2013). Novel, low cost, highly effective, handmade steroid pellets for experimental studies. PloS One 8:e64049. doi: 10.1371/journal.pone.0064049, PMID: 23691144 PMC3655057

[ref32] Sarkola T. Mäkisalo H. Fukunaga T. Eriksson C. J. P. (1999). Acute effect of alcohol on estradiol, Estrone, progesterone, prolactin, cortisol, and luteinizing hormone in premenopausal women. Alcohol. Clin. Exp. Res. 23, 976–982. doi: 10.1111/j.1530-0277.1999.tb04215.x, PMID: 10397281

[ref33] Satta R. Hilderbrand E. R. Lasek A. W. (2018). Ovarian hormones contribute to high levels of binge-like drinking by female mice. Alcohol. Clin. Exp. Res. 42, 286–294. doi: 10.1111/acer.13571, PMID: 29205408 PMC5785425

[ref34] Seif T. Chang S. J. Simms J. A. Gibb S. L. Dadgar J. Chen B. T.. (2013). Cortical activation of accumbens hyperpolarization-active NMDARs mediates aversion-resistant alcohol intake. Nat. Neurosci. 16, 1094–1100. doi: 10.1038/nn.3445, PMID: 23817545 PMC3939030

[ref35] Shanley M. R. Miura Y. Guevara C. A. Onoichenco A. Kore R. Ustundag E.. (2023). Estrous cycle mediates midbrain neuron excitability altering social behavior upon stress. J. Neurosci. 43, 736–748. doi: 10.1523/JNEUROSCI.1504-22.2022, PMID: 36549906 PMC9899085

[ref36] Sneddon E. A. Fennell K. A. Bhati S. Setters J. E. Schuh K. M. DeMedio J. N.. (2023a). Greater resistance to footshock punishment in female C57BL/6J mice responding for ethanol. Alcohol. Clin. Exp. Res. 47, 678–686. doi: 10.1111/acer.15039, PMID: 36822578 PMC10149597

[ref37] Sneddon E. A. Masters B. M. Ream K. D. Fennell K. A. DeMedio J. N. Cash M. M.. (2023b). Sex chromosome and gonadal hormone contributions to binge-like and aversion-resistant ethanol drinking behaviors in four Core genotypes mice. Front. Psych. 14:322. doi: 10.3389/fpsyt.2023.1098387 PMC10027717 36960454

[ref38] Sneddon E. A. Masters B. M. Shi H. Radke A. K. (2023c). Removal of the ovaries suppresses ethanol drinking and promotes aversion-resistance in C57BL/6J female mice. Psychopharmacology (Berl). 1–10. doi: 10.1007/s00213-023-06456-x 37653347 PMC11170684

[ref39] Sneddon E. A. Ramsey O. R. Thomas A. Radke A. K. (2020). Increased responding for alcohol and resistance to aversion in female mice. Alcohol. Clin. Exp. Res. 44:1400. doi: 10.1111/acer.14384 32472651

[ref40] Sneddon E. A. Rasizer L. N. Cavalco N. G. Jaymes A. H. Ostlie N. J. Minshall B. L.. (2022). Gonadal hormones and sex chromosome complement differentially contribute to ethanol intake, preference, and relapse-like behaviour in four core genotypes mice. Addict. Biol. 27:e13222. doi: 10.1111/adb.13222, PMID: 36001422 PMC9413386

[ref41] Sneddon E. A. White R. D. Radke A. K. (2019). Sex differences in binge-like and aversion-resistant alcohol drinking in C57BL/6J mice. Alcohol. Clin. Exp. Res. 43, 243–249. doi: 10.1111/acer.13923 30431655

[ref42] Ström J. O. Theodorsson A. Ingberg E. Isaksson I. M. Theodorsson E. (2012). Ovariectomy and 17β-estradiol replacement in rats and mice: a visual demonstration. J. Vis. Exp. 7:4013. doi: 10.3791/4013 PMC3471296 22710371

[ref44] Torres O. V. Walker E. M. Beas B. S. O’Dell L. E. (2014). Female rats display enhanced rewarding effects of ethanol that are hormone dependent. Alcohol. Clin. Exp. Res. 38, 108–115. doi: 10.1111/acer.12213, PMID: 23909760 PMC3842413

[ref45] Vandegrift B. J. Hilderbrand E. R. Satta R. Tai R. He D. You C.. (2020). Estrogen receptor α regulates ethanol excitation of ventral tegmental area neurons and binge drinking in female mice. J. Neurosci. 40, 5196–5207. doi: 10.1523/JNEUROSCI.2364-19.2020 32482639 PMC7329299

[ref46] Vandegrift B. J. You C. Satta R. Brodie M. S. Lasek A. W. (2017). Estradiol increases the sensitivity of ventral tegmental area dopamine neurons to dopamine and ethanol. PloS One 12:e0187698. doi: 10.1371/journal.pone.0187698 29107956 PMC5673180

[ref47] Weinland C. Mühle C. Kornhuber J. Lenz B. (2021). Progesterone serum levels correlate negatively with craving in female postmenopausal in-patients with alcohol use disorder: a sex- and menopausal status-separated study. Prog Neuro-Psychopharmacology Biol Psychiatry 110:110278. doi: 10.1016/j.pnpbp.2021.110278, PMID: 33571605

[ref001] White A. M. (2020). Gender differences in the epidemiology of alcohol use and related harms in the United States. Alcohol research: current reviews. 40. 10.35946/arcr.v40.2.01 PMC7590834 33133878

[ref48] Xie Q. Buck L. A. Bryant K. G. Barker J. M. (2019). Sex differences in ethanol reward seeking under conflict in mice. Alcohol. Clin. Exp. Res. 43, 1556–1566. doi: 10.1111/acer.14070, PMID: 31034618 PMC6602812

